# 18F-FDG Positron Emission Tomography – An Innovative Technique for the Diagnosis of a Canine Lameness

**DOI:** 10.3389/fvets.2016.00045

**Published:** 2016-06-08

**Authors:** Kelly Mann, Juliette Hart, Felix Duerr

**Affiliations:** ^1^Veterinary Diagnostic Imaging Section, Department of Environmental and Radiological Health Sciences, Colorado State University, Fort Collins, CO, USA; ^2^Small Animal Sports Medicine and Rehabilitation Section, Department of Clinical Sciences, Colorado State University, Fort Collins, CO, USA

**Keywords:** positron emission tomography, computed tomography, fluorodeoxyglucose, canine, lameness

## Abstract

**Introduction:**

Positron emission tomography (PET) imaging with fluorine-18-fluorodeoxyglucose (18F-FDG) is widely known for its use in the diagnosis and tracking of primary and metastatic tumors *via* uptake and retention of the radiopharmaceutical by hypermetabolic cells. 18F-FDG is also used to study the normal physiology of glucose uptake, metabolism, and muscle activity during and after exercise.

**Background:**

A pilot study adding PET imaging to the diagnostic evaluation of canine patients undergoing computed tomography (CT) for mild or intermittent thoracic and pelvic limb lameness is ongoing. Dogs with an observable (grade 1–2/5) lameness that have undergone routine radiography and complete physical examination by board-certified veterinary surgeons and sports medicine and rehabilitation specialists are enrolled. Each patient undergoes leash walking for 15 min prior to premedication and induction of general anesthesia for the PET–CT examination. 18F-FDG is injected intravenously, and a whole-body PET examination is conducted after 1 h of radiopharmaceutical uptake time. Standard algorithm, whole-body pre- and post-contrast CT examinations, and focused, standard, and bone algorithm CT scans of the thoracic or pelvic limb areas of interest are obtained concurrently. Abnormal PET–CT findings are further investigated with additional diagnostic imaging or at surgery (e.g., ultrasound, MRI, and arthroscopy).

**Discussion:**

This case report uses a canine patient referred for thoracic limb lameness to illustrate the role of advanced imaging in a diagnostic plan and to discuss a recommended PET–CT procedure for lameness evaluation. The PET–CT imaging protocol recommended in this report was designed to significantly enhance a routine thoracic limb CT examination and to identify areas of muscle, tendon, or ligament overuse, inflammation, or injury for further diagnostic procedures or definitive treatment.

**Concluding remarks:**

18F-FDG PET–CT adds valuable physiologic and anatomic information to the diagnostic evaluation of patients presenting with indistinct or intermittent clinical signs of musculoskeletal inflammation or injury. In addition, tailoring the PET acquisition and radiopharmaceutical parameters allows for detailed information gathering to more closely assess normal and abnormal physiology, unlocking a new frontier in the study of canine athletic injury and optimal performance.

## Introduction

An 8.5-year-old castrated male, 20.9 kg, Chow-mix dog was presented to the Small Animal Sports Medicine and Rehabilitation Service for evaluation of a mild, intermittent right thoracic limb lameness of approximately 1-year duration. The patient history includes hip dysplasia with mild degenerative joint disease of both coxofemoral joints and grade 1–2/4 left-sided systolic heart murmur. Previous radiographs of the right shoulder and elbow were normal, and routine laboratory tests of blood and urine were also within normal limits. On orthopedic examination, he had a grade 1/5 right thoracic limb lameness at the trot and intermittent discomfort on Campbell’s test with normal range of motion and no joint effusion. Campbell’s test is based on a study evaluating collateral ligament integrity by supinating and pronating the carpus when the elbow and carpus are flexed in a 90° position (1). Modifications also include applying gentle pressure to the medial compartment and subjectively assessing patient reaction (2). The patient also had mild thickening at the distal insertion of the right flexor carpi ulnaris (FCU) tendon.

Challenging orthopedic and sports medicine cases are often referred for advanced imaging after routine diagnostic procedures are performed by the referring veterinarian. Magnetic resonance imaging (MRI) and computed tomography (CT) are frequently the next imaging procedures of choice. At our institution, a pilot study adding positron emission tomography (PET) to the diagnostic evaluation of canine patients undergoing CT as part of the normal diagnostic plan for mild or intermittent thoracic or pelvic limb lameness is ongoing. On the morning of the PET–CT examination, the patient was leash-walked for 15 min prior to premedication and induction of general anesthesia. The fluorine-18-fluorodeoxyglucose (18F-FDG) was injected intravenously, and a whole-body PET examination was conducted after 1 h of radiopharmaceutical uptake time. Increased radiopharmaceutical uptake (IRU) was identified in the FCU muscle of the right antebrachium, and a slight increase in muscle size and density (approximately 20 HU) was noted in corresponding CT images. A faint ring of contrast enhancement corresponding to a 5-mm area of the most intense radiopharmaceutical uptake was also noted. A diagnostic ultrasound examination performed the following day revealed a hypoechoic region consistent with muscle tearing within the right FCU muscle. Patient discharge instructions included: daily stretching of the thoracic limb muscles, restricted activity within the home (e.g., no running, jumping, stairs, or rough play), leash walks of no more than 10 min duration for 6 weeks, and a NSAID (37.5 mg Carprofen *per os* every 12 h) as needed for pain relief. After 6 weeks, the length of daily walks was gradually increased 5–10 min per week until the patient could comfortably walk for 30 min without evidence of lameness. A recheck ultrasound examination was performed 6 weeks after discharge, and an injection of platelet rich plasma (PRP) was performed at that time. Additional ultrasound and physical examinations are planned at 3-month intervals.

This case report uses a canine patient referred for a subtle forelimb lameness to illustrate the role of advanced imaging in a diagnostic plan and to recommend an innovative PET–CT imaging protocol, which identifies glucose uptake abnormalities and asymmetries useful in the diagnosis of muscular overuse, inflammation, or infection, in clinically lame canine patients.

## Background

Positron emission tomography imaging with 18F-FDG is widely known for its use in the diagnosis and tracking of primary and metastatic tumors *via* uptake and retention of the radiopharmaceutical by hypermetabolic cells (3). 18F-FDG is also used to study the physiology of glucose uptake, metabolism, and muscle activity during and after exercise (4, 5) or as a result of concurrent disease conditions (6, 7). The additional physiologic data gained from PET imaging are of particular interest to the Sports Medicine and Rehabilitation Service because they may aid in the diagnosis of subtle abnormalities that are not apparent on standard radiographic or US techniques. Additionally, it is not feasible to perform whole-body scanning with US or MR modalities, and similar to its extensive use in oncology, PET–CT can provide objective morphologic lesion localization and quantifiable functional measurements to document response to therapeutic intervention in muscle groups that are difficult to evaluate through other means. Availability of integrated PET–CT equipment has increased in veterinary university and referral centers because of its universal application in cancer diagnosis and response to therapy. Although the half-life of fluorine-18 is short (110 min), commercial production for human medical procedures is common, which increases the logistic availability of the radiopharmaceutical, and the short half-life allows release of the patient on the same day. In our geographic area, the availability and cost of 18F-FDG is reasonable because of its extensive use in human and veterinary oncology. Its use in human musculoskeletal diagnostic imaging and research is also increasing. Canine patients are commonly referred to our institution for shoulder and elbow pathology but also for lumbar spine or pelvic limb pain, creating the opportunity for PET as a complementary procedure, which adds physiological data to the CT findings.

## Equipment

Positron emission tomography–computed tomography imaging was performed with a Philips GEMINI TF Big Bore PET–CT system (Philips Healthcare, Koninklijke Philips Electronics N.V., Amsterdam). This system combines a 16-slice CT machine with time-of-flight (TOF) PET technology capable of 495-ps timing resolution and list mode reconstruction. The PET and CT gantries have an 85-cm inner diameter with up to 60-cm diameter field of view.

## PET–CT Imaging Protocol

Immediately prior to premedication and induction of general anesthesia, the patient is leash-walked for a minimum of 15 min. Patient monitoring during the anesthetic episode includes: ECG, arterial pressure, capnography, pulse oximetry, ventilation rate and quality, and urinary catheterization. The patient is positioned on the PET–CT table in dorsal recumbency, and the table position is zeroed. 18F-FDG (5.2–6.3 MBq/kg) is injected *via* indwelling catheter in the cephalic or saphenous vein. The venous and arterial catheter locations are selected to specifically avoid the thoracic or pelvic limb area of interest noted in the patient history. Local radiation safety policies require that the 1-h radiopharmaceutical uptake time occurs in the PET–CT suite. After intravenous administration of the radiopharmaceutical, the patient’s vital signs and plane of anesthesia are monitored remotely from the shielded PET–CT control room.

At 20 min prior to PET acquisition (after 40 min of uptake time), a non-contrast helical CT series focused on the thoracic or pelvic limb area of interest is performed and reconstructed with 1-mm bone and 2-mm standard filters in a 1024 × 1024 matrix. A non-contrast whole-body CT series immediately follows the focused series, and 5- and 2-mm (768 × 768 matrix) standard reconstructions are created.

After 60 min of radiopharmaceutical uptake time, a whole-body PET acquisition using consecutive 8-cm beds with 1.5-min bed times is initiated. At the conclusion of the PET acquisition, an early venous phase contrast-enhanced whole-body CT is performed using a standard algorithm, with 5- and 2-mm standard reconstructions as described in the non-contrast series above. For the contrast-enhanced CT, 2 ml/kg of a non-ionic, iodinated contrast solution [iohexol 755 mg/ml (equivalent to 350 mg of organic iodine), GE Healthcare] is administered *via* autoinjector at 2 ml/s followed by 1 ml/kg of 0.9% NaCl at 2 ml/s. The CT scan begins after a 40- to 50-s delay based on patient heart rate.

## Post-Scanning Procedures

All images from each of the PET and CT acquisitions are transferred to the hospital picture archiving and communication system and fused PET–CT images are viewed on a dedicated Philips IntelliSpace Portal workstation (v6.0.2.32500, 13 June 2014, Koninklijke Philips Electronics N.V.) for clinical interpretation. In our experience, the blue palette (cardiac) lookup table (LUT) significantly increases the conspicuity of radiopharmaceutical uptake in fused PET–CT images when compared to the red palette (thermal) LUT. The patient recovers from anesthesia in a dedicated nuclear medicine ward and segregated walking area with closed circuit television monitoring after extubation. Local radiation safety policies allow the patient to return to the client or other sections of the veterinary teaching hospital after reaching a survey meter reading of ≤20 μSv/h at the skin surface of the shoulder region. Abnormal PET–CT findings are further investigated with additional diagnostic imaging or at surgery (e.g., ultrasound, MRI, and arthroscopy).

## Discussion

**Figure 1:**
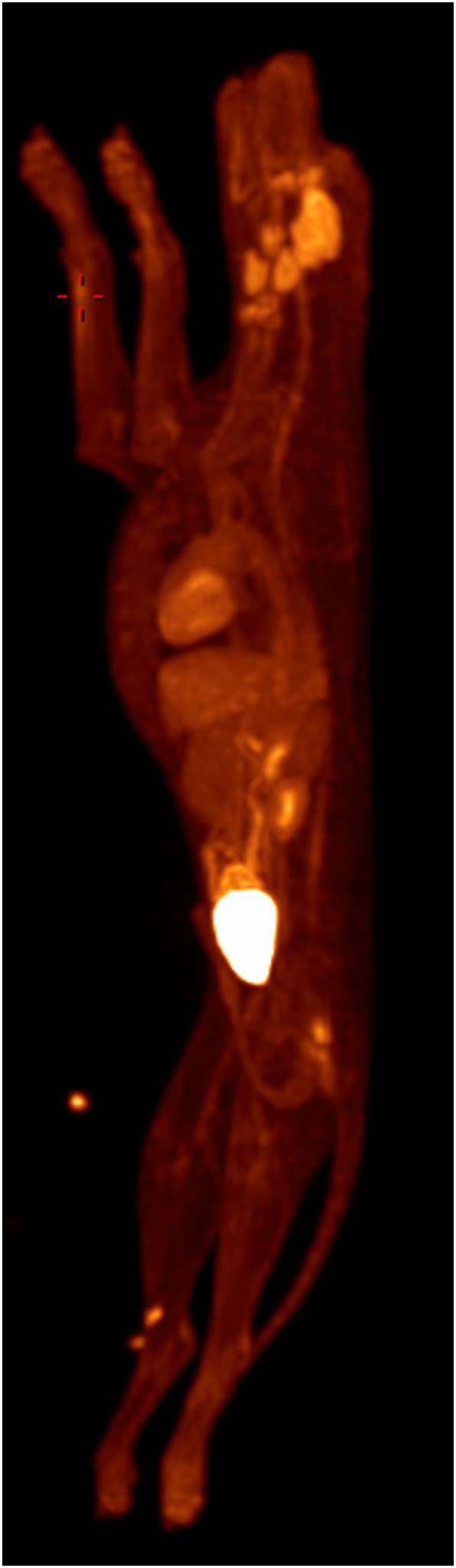
**Whole-body PET MIP with abnormal 18F-FDG uptake noted in right thoracic limb (red crosshairs)**. Normal radiopharmaceutical uptake in the brain, zygomatic and mandibular salivary glands, vocal folds, myocardium, abdominal viscera, and urinary tract is also present.

The initial clinical observations confirmed a chronic, mild right thoracic limb lameness, but failed to localize the specific cause. A PET–CT was recommended and elected. Fused PET–CT images were evaluated in multiple, preset, and adjustable Hounsfield unit window levels and widths to identify abnormal areas of uptake or areas of asymmetric uptake in the skeletal muscles of the thoracic limbs, pelvic limbs, or axial regions of the patient. The three-dimensional PET maximum intensity projection (MIP) is helpful for orientation prior to detailed review of the data acquisitions in two-dimensional transverse, sagittal, and dorsal planes or three-dimensional CT volume rendering (Figure 1).

Setting the PET overlay to display a narrow standardized uptake value (SUV) window width (0–5) is particularly important when searching for asymmetric radiopharmaceutical uptake in skeletal muscle because subtle areas of increased uptake must be conspicuous in comparison to the surrounding or contralateral normal tissues.

**Figure 2:**
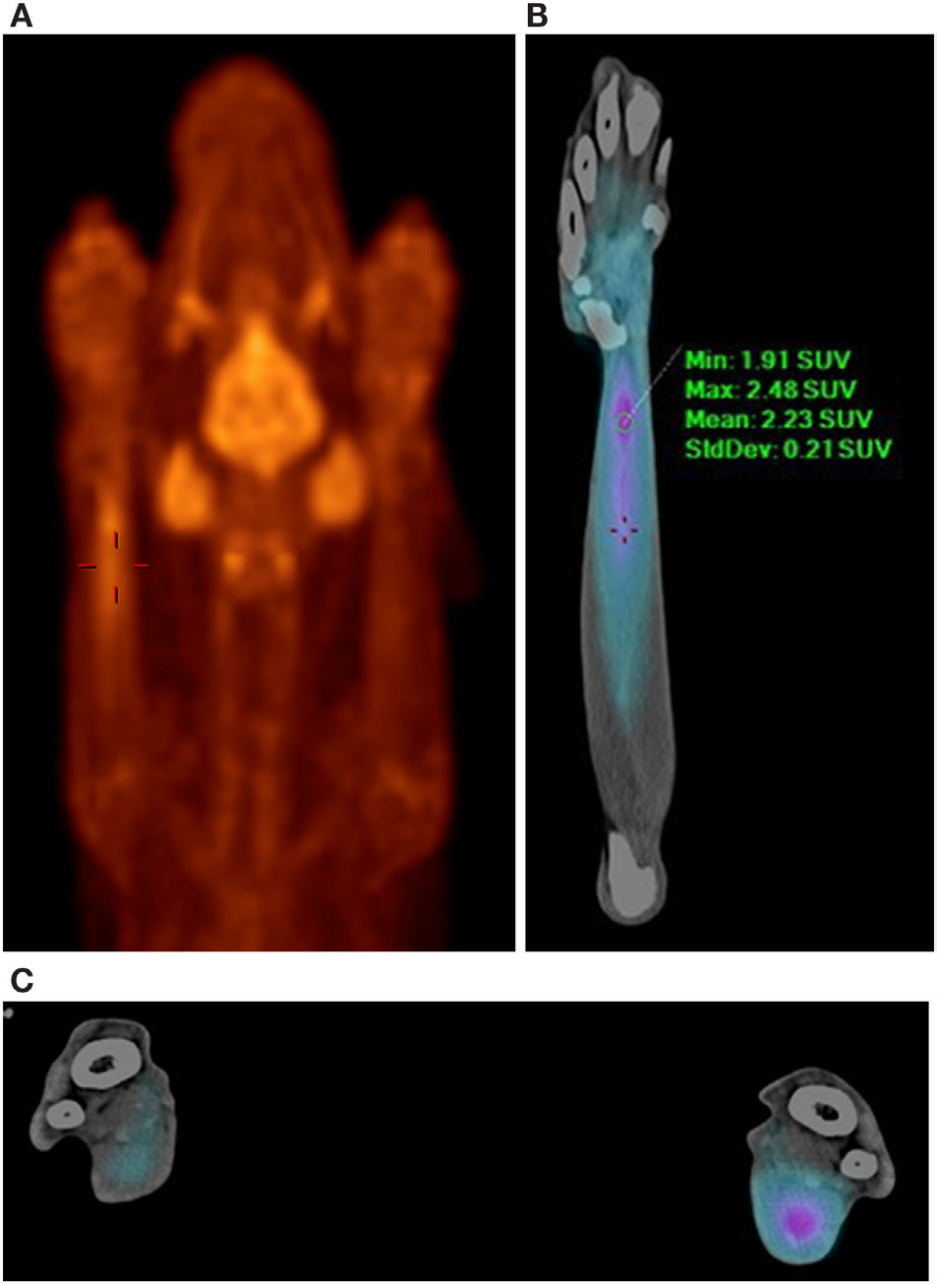
**PET–CT images demonstrating increased 18F-FDG uptake in the right flexor carpi ulnaris muscle**. **(A)** PET MIP, ventral view, red palette (thermal) lookup table (LUT). Normal radionuclide uptake is also present in the brain, zygomatic and mandibular salivary glands, and vocal folds. **(B)** PET–CT, ventral view, right antebrachium, blue palette (cardiac) LUT. **(C)** PET–CT, transverse view, blue palette (cardiac) LUT. Comparison of the right and left antebrachii at the level of the right flexor carpi ulnaris muscle injury. The right antebrachium is on the right of the image. The blue palette LUT increases lesion conspicuity when viewing fused images.

**Figure 3:**
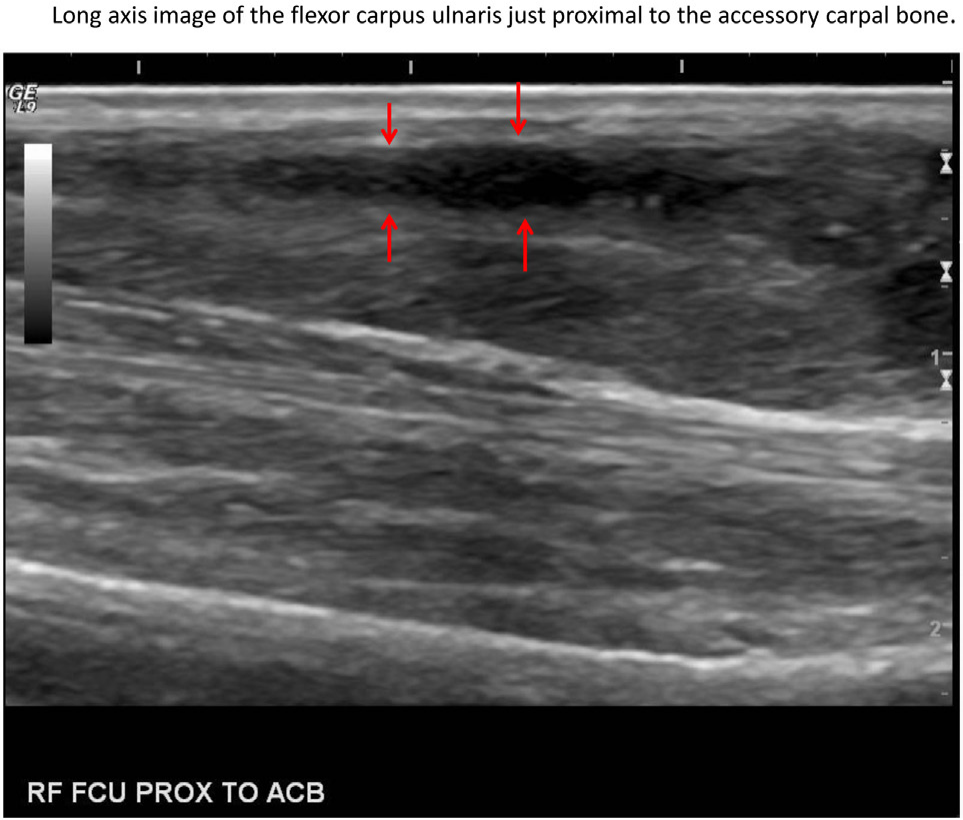
**Sagittal ultrasound image of the muscle tear (red arrows) in the right flexor carpi ulnaris muscle just proximal to the accessory carpal bone (left side of image is distal)**.

In this case, normal uptake of 18F-FDG was noted within the central nervous system, zygomatic and mandibular salivary glands, vocal folds, myocardium, and abdominal viscera, as well as normal excretion *via* the urinary tract (8–10) (Figure 1). On evaluation of the radionuclide uptake within the muscles of the thoracic limbs, asymmetrically IRU was identified in the FCU muscle of the right antebrachium with a maximum standardized uptake value (SUVmax) of 2.4, compared to the contralateral muscle with 1.0 SUVmax (11) (Figures 2A–C). This finding is related to increased cellular metabolic activity and may be the result of compensatory overuse of the muscle, inflammation, or injury. IRU in a neoplastic cell results from unregulated increase in glucose transporters and dependence on glycolysis for energy production. Inflammation, injury, or increased use of glucose by non-neoplastic muscle cells causes IRU secondary to normal physiologic glycolysis in response to cell injury and repair mechanisms. Although iodinated contrast may increase the conspicuity of soft tissue injury in CT images as a result of neovascularization or extravasation from increased vascular permeability, a change in the density, size, or enhancement may be inconspicuous or mild, as in this patient (12). Contrast enhancement and tissue density in CT images are affected by variability in the amount of neovascularization, vascular permeability and edema in the lesion itself, and the timing of contrast administration. In this case, a diagnostic ultrasound examination identified two small hypoechoic areas within the muscle of the right FCU, just proximal to the insertion on the accessory carpal bone. Corresponding irregular and hypoechoic muscle fibers were also visible on long axis images (Figure 3). These findings were consistent with muscle tearing within the right FCU muscle and corresponded to the most intense region of radiopharmaceutical uptake on the PET–CT images (Figure 2B) (13, 14). The extent of IRU identified a larger area of affected FCU muscle, when compared to the small focus of CT contrast enhancement. At the recheck ultrasound examination 6 weeks after discharge, an intralesional injection of platelet-rich plasma (PRP) was performed to promote healing. Clinical use of PRP is becoming commonplace in both human and veterinary medicine for the treatment of musculoskeletal injuries. The beneficial effects of PRP are attributed in part to growth factors released by activated platelets leading to neovascularization (15–18). Additional ultrasound and physical examinations to document the healing process are scheduled every 3 months.

There are unique challenges to overcome when conducting PET–CT imaging of veterinary patients, and attention to certain details can maximize the quality of the data acquisition. A short period of mild muscular effort (leash walking) is recommended prior to anesthesia to increase the possibility of detecting asymmetric radiopharmaceutical uptake in individual muscles or muscle groups. However, strenuous muscle activity (e.g., focused strength training, sled pulling, or excessive physical effort) can result in IRU for 24–48 h after the event and upset the balance between detectable asymmetry versus induced normal physiologic uptake that masks underlying abnormal pathologic uptake. External monitoring instrumentation and intravenous and intra-arterial catheters are placed outside the thoracic or pelvic limb area of interest to avoid the possibility of beam hardening artifacts or IRU from inflammation or extravasation at the site of medication, iodinated contrast, or radiopharmaceutical injection. Using radiolucent, padded v-troughs, wedges, and blocks to position the patient in dorsal recumbency, with thoracic limbs moderately extended, elbows and paws parallel to one another, and pelvic limbs extended with stifles, and paws parallel to one another will help to achieve bilateral symmetry. This also extends the spine axially to avoid curvature and allow better evaluation of the cervical and lumbosacral regions. Positioning the patient with the pelvic limbs first into the gantry allows the greatest access to the endotracheal tube, long ventilation tubes, anesthesia cart, and patient monitoring display screens (remotely viewed on closed circuit or wireless monitors in the CT control room) as the patient table travels more than 2 m in and out of the PET and CT gantries. This consistent and symmetric method of positioning also greatly assists in the assessment of ipsilateral and contralateral bone and soft tissue anatomical structures during image evaluation, improving comparison and detection of abnormalities of radiopharmaceutical uptake, iodinated contrast enhancement, density, and size. An indwelling urinary catheter minimizes urinary bladder size, improving image interpretation in the caudal abdomen, and facilitates the removal of radioactive urine, protecting against contamination of the gantry and further decreasing patient exposure.

This PET–CT protocol is also consistent with the tenets of radiation safety and published nuclear medicine dosing recommendations. Local radiation safety policies require the radionuclide uptake time to occur in the PET–CT suite. All personnel wear body and ring badges, lab coats, gloves, and closed-toe shoes. The patient achieves a stable plane of general anesthesia prior to radiopharmaceutical administration, and remote monitoring equipment minimizes contact with the patient during the PET–CT data acquisition and anesthesia recovery time periods. The veterinary patient dose is in accordance with North American and European Association of Nuclear Medicine recommendations for human pediatric patients, further ensuring that the exposure to veterinary personnel will be minimized (19).

## Concluding Remarks

Integrated PET–CT is a significant diagnostic method, synergizing the localization and anatomic and physiologic data analysis tools of two advanced imaging modalities. Although primarily known for extensive use in the field of oncology, the application of 18F-FDG imaging for the clinical evaluation and research of musculoskeletal disease conditions and normal exercise physiology is increasing. The PET–CT imaging protocol recommended in this report was designed to significantly enhance a routine thoracic or pelvic limb CT examination and to identify areas of muscle, tendon, or ligament overuse, inflammation, or injury for further diagnostic procedures or definitive treatment. The 18F-FDG PET–CT added valuable physiologic and anatomic information to the diagnostic evaluation of this patient, which can also be used for objective evaluation of the healing process in serial examinations. Current efforts are aimed at the validation of this technique through demonstrated benefit to additional patients in the ongoing pilot study. PET–CT imaging is recommended after standard means of diagnosis have failed or when the specific physiologic information provided by a PET radiopharmaceutical may detect subtle metabolic abnormalities in the muscle, indicating compensatory overuse, inflammation, or injury. Continued tailoring of the PET acquisition and radiopharmaceutical parameters will allow for more detailed information gathering in the realm of exercise physiology. Future efforts will increase the objective PET–CT data available to more closely assess normal and abnormal physiology, unlocking a new frontier in the study of canine athletic injury and optimal performance.

## Author Contributions

This case report was designed and coordinated by KM, JH and FD to specifically introduce a novel imaging protocol and the reasoning behind its use in the diagnostic evaluation of canine patients with intermittent or subtle, undiagnosed lameness.

## Conflict of Interest Statement

The authors declare that the research was conducted in the absence of any commercial or financial relationships that could be construed as a potential conflict of interest.

## References

[1] Campbell JR Nonfracture injuries to the canine elbow. J Am Vet Med Assoc (1969) 155(5):735–44. 5257293

[2] Farrell M Draffan D Gemmill T Mellor D Carmichael S. In vitro validation of a technique for assessment of canine and feline elbow joint collateral ligament integrity and description of a new method for collateral ligament prosthetic replacement. Vet Surg (2007) 36:548–56. 10.1111/j.1532-950X.2007.00281.x 17686128

[3] Alazraki N Shumate M Kooby D FDG PET and PET/CT molecular imaging. In: A Clinician’s Guide to Nuclear Oncology: Practical Molecular Imaging and Radionuclide Therapies. Reston, VA: The Society of Nuclear Medicine (2007). p. 1–6.

[4] Rudroff T Kalliokoski KK Block DE Gould JR Klingensmith WC III Enoka RM. PET/CT imaging of age- and task-associated differences in muscle activity during fatiguing contractions. J Appl Physiol (2013) 114:1211–9. 10.1152/japplphysiol.01439.2012 23412899 PMC3656430

[5] Rudroff T Kindred JH Kalliokoski KK [18F]-FDG positron emission tomography – an established tool opening a new window into exercise physiology. J Appl Physiol (2015) 118:1181–90. 10.1152/japplphysiol.01070.2014 25767034

[6] Aydin A Hickeson M Yu JQ Zhuang H Alavi A. Demonstration of excessive metabolic activity of thoracic and abdominal muscles on FDG-PET in patients with chronic obstructive pulmonary disease. Clin Nucl Med (2005) 30(3):159–64. 10.1097/00003072-200503000-00003 15722818

[7] Rudroff T Kindred JH Koo PJ Karki R Hebert JR. Asymmetric glucose uptake in leg muscles of patients with multiple sclerosis during walking detected by [18F]-FDG PET/CT. NeuroRehabilitation (2014) 35(4):813–23. 10.3233/NRE-141179 25323085

[8] LeBlanc A Jakoby B Townsend D Daniel G. Thoracic and abdominal organ uptake of 2-deoxy-2-[18F]fluoro-d-glucose (18FDG) with positron emission tomography in the normal dog. Vet Radiol Ultrasound (2008) 49(2):182–8. 10.1111/j.1740-8261.2008.00348.x 18419002

[9] Lee M Lee A Jung M Lee I Choi J Chung H Characterization of physiologic 18F-FDG uptake with PET-CT in dogs. Vet Radiol Ultrasound (2010) 51(6):670–3. 10.1111/j.1740-8261.2010.01727.x 21158245

[10] Randall E Loeber S Kraft S Physiologic variants, benign processes, and artifacts from 106 canine and feline FDG-PET/Computed Tomography scans. Vet Radiol Ultrasound (2013) 55(2):213–26. 10.1111/vru.12138 24467373

[11] Lee K Basic science of nuclear medicine. In: Bailey DL Townsend DW Valk PE Maisey MN, editors. Positron Emission Tomography. Reston, VA: Society of Nuclear Medicine and Molecular Imaging (2015). p. 167–88.

[12] Rossmeisl JH Rohleder JJ Hancock R Lanz OI. Computed tomographic features of suspected traumatic injury to the iliopsoas and pelvic limb musculature of a dog. Vet Radiol Ultrasound (2004) 45(5):388–92. 10.1111/j.1740-8261.2004.04070.x 15487562

[13] Bohndorf K Kilcoyne RF. Traumatic injuries: imaging of peripheral musculoskeletal injuries. Eur Radiol (2002) 12:1605–16. 10.1007/s00330-002-1461-8 12111052

[14] Mattoon JS Nyland TG, editors. Musculoskeletal system. 3rd ed Small Animal Diagnostic Ultrasound. (Chap. 14), St. Louis, MO: Saunders (2015). p. 517–40.

[15] Visser LC Arnoczky SP Caballero O Kern A Ratcliffe A Gardner KL. Growth factor-rich plasma increases tendon cell proliferation and matrix synthesis on a synthetic scaffold: an in vitro study. Tissue Eng (2010) 16(3):1021–9. 10.1089/ten.TEA.2009.0254 19839921

[16] Bosch G Moleman M Barneveld A van Weeren PR van Schei HTM. The effect of platelet-rich plasma on the neovascularization of surgically created equine superficial digital flexor tendon lesions. Scand J Med Sci Sports (2011) 21:554–61. 10.1111/j.1600-0838.2009.01070.x 20459479

[17] Visser LC Arnoczky SP Caballero O Gardner KL Evaluation of the use of an autologous platelet rich fibrin membrane to enhance tendon healing in dogs. Am J Vet Res (2011) 72(5):699–705. 10.2460/ajvr.72.5.699 21529224

[18] Beitzel K Allen D Apostolakos J Russell R McCarthy MB Gallo G US definitions, current use, and FDA stance on use of platelet-rich plasma in sports medicine. J Knee Surg (2015) 28:29–34. 10.1055/s-0034-1390030 25268794

[19] Lassmann M Treves ST. Paediatric radiopharmaceutical administration: harmonization of the 2007 EANM paediatric dosage card (version 1.5.2008) and the 2010 North American consensus guidelines. Eur J Nucl Med Mol Imaging (2014) 41(5):1036–41. 10.1007/s00259-014-2731-9 24599377

